# Association between dating app use and unhealthy weight control behaviors and muscle enhancing behaviors in sexual minority men: a cross-sectional study

**DOI:** 10.1186/s12889-023-15715-7

**Published:** 2023-05-09

**Authors:** Alvin Tran, Mabintou Darboe, Anirudh Goyal, Nick Birk

**Affiliations:** 1 Department of Population Health and Leadership, School of Health Sciences, University of New Haven, 300 Boston Post Road, West Haven, CT 06516 USA; 2 Harvard T.H. Chan School of Public Health, 67 Huntington Ave, Boston, MS, MA 02115 USA

**Keywords:** Dating apps, Unhealthy weight control behaviors, Muscle enhancing behaviors, Sexual minority men, Body image

## Abstract

**Background:**

Body image concerns are prevalent and are viewed as risk factors for engaging in unhealthy weight control behaviors (UWCBs), such as purging, fasting, and the misuse of laxatives and diet pills. Studies have also linked UWCBs to the development of eating disorders. In the United States (U.S.), sexual minority men (e.g., bisexual, gay, and men who have sex with men) are prone to UWCBs often as a result of societal pressures to achieve certain standards of appearance – of which are often perpetuated through various media platforms. A growing number of studies have explored the possible role mobile dating applications (“dating apps”) play in contributing to UWCBs. To our knowledge, such studies have not explored this possible relationship between dating apps and UWCBs in sexual minority men (SMM). To fill this gap in the scientific literature, the present study assessed the association between dating app use and UWCBs and muscle enhancing behaviors among a sample of adult SMM in the U.S.

**Method:**

549 SMM participated in an anonymous survey from Qualtrics Survey Panels. UWCBs and muscle enhancing behaviors were assessed through items adapted from national surveys. Body image dissatisfaction was assessed using the Male Body Attitudes Scales. Participants also reported their history of dating app use. We performed descriptive statistics, chi-square tests, and student’s t-tests. Multivariable logistic regression models assessed the relationship between dating app use and UWCBs and muscle enhancing behaviors.

**Results:**

Dating app users had significantly higher body image dissatisfaction scores than non-users. Dating app users also demonstrated significantly elevated odds of engaging in four UWCBs and muscle enhancing behaviors: laxatives, diet pills, muscle-building supplements, and protein powders.

**Conclusions:**

This is one of the first studies to assess dating app use and its association with UWCBs and muscle enhancing behaviors in SMM. Increased surveillance and detection for such behaviors among SMM, particularly those using dating apps, are needed.

**Supplementary Information:**

The online version contains supplementary material available at 10.1186/s12889-023-15715-7.

## Introduction

In contemporary culture, thinness and a so called “fit” physique are often emphasized as the ideal body size and shape for men and women. For men, this ideal body image is often characterized as mesomorphic – possessing high levels of muscle mass [1, 2]. For sexual minority men (SMM), this ideal body image is further described as muscular, thin, and often light-skin toned [3]. The strive to achieve this ideal physique may promote many individuals to engage in risky and unsafe behaviors to lose weight, gain muscle, and make other changes to their body shape and size. Some engage in unhealthy weight control behaviors (UWCBs) ranging from purging and fasting to the use of laxatives and diet pills [4–7]. UWCBs are of great concern as studies have documented their association with the development of depression and eating disorders, including bulimia nervosa and anorexia nervosa [6, 8–11].

Research continues to document bisexual and gay males globally are increasingly engaging in UWCBs. According to two literature reviews assessing differences across sexual orientation identity, bisexual and gay males were more likely to engage in UWCBs than their heterosexual/straight male counterparts [12, 13]. Furthermore, in a study assessing the prevalence of UWCBs among nearly 13,000 individuals, Hadland et al. [14], found sexual minority males demonstrated significantly higher odds of engaging in UWCBs than heterosexual/straight males. The high prevalence of UWCBs among SMM, including bisexual and gay men, is often attributed to stereotypes and social stigma about physical appearance in the dominant gay community [12]. In particular, research findings have described the ideal male appearance in dominant gay culture as one with a muscular and thin body shape and size [3, 12]. Many gay and bisexual men may feel pressured to conform to such high appearance standards and develop body dissatisfaction and engage in UWCBs as a result. For example, Parker and Harriger’s literature review [12] found SMM experience greater body dissatisfaction and greater desire for muscularity; they also demonstrated greater desire to look like the men they see in the media [12, 15, 16].

Body image dissatisfaction has been identified as a key risk factor for UWCBs [8, 9, 12, 17, 18]. According to the Tripartite Influence Model of body image, factors such as parents, peers, and the media contribute to body image dissatisfaction through internalization and comparison of appearance ideals [19]. The media, in particular, is reported to have more influence on the development of body image dissatisfaction [20]. In a study to determine the influence of social media platforms and dating applications on body dissatisfaction among 2,733 SMM, Griffiths et al. [18] found that media platforms based on sharing pictures (image-centric) increased eating disorder behaviors and muscularity dissatisfaction. Other studies have also found image-centric social media to be associated with greater body image dissatisfaction and disordered eating behaviors among SMM [21, 22].

Much like image-centric social media platforms, online dating applications, also known as “dating apps” in popular culture, may pose a similar threat to SMM’s body image dissatisfaction [23, 24]. Dating apps, a specific form of social media, often provide an online platform where users can identify potential romantic and sexual partners based primarily on images and short profiles [24, 25]. The dating app known as Tinder, for example, allows its users to match with others by swiping right (to express one’s interest in another user) or left (to express one’s disinterest) on profiles that generally have photos, including headshots [25]. Other dating apps have similar features where users can “tap” (e.g., Grindr) and “like” (e.g., Hinge) images to express interest [26, 27]. These methods of selecting romantic and sexual partners have been associated with an increased desire to embrace societal body image ideals among SMM [23, 25]. Moreover, users of dating apps often posts and share profile pictures, including ones that are digitally altered, that depict ideal body images; such images often render dating app users to internalize and compare themselves with unrealistic appearance standards, increasing body image dissatisfaction and UWCB engagement among users [24]. It is important to also highlight that there are other uses of dating apps beyond searching for romantic and sexual interests. Other uses include psychosocial motivations, such as the thrill of excitement that comes with meeting people through the apps and validating one’s self-worth through the receiving of positive feedback [25].

The number of SMM using dating apps has extensively increased over the years [24, 28]. For example, Grindr, a dating app marketed to SMM, had approximately 13 million monthly users in 2020 compared to when it was created in 2009 with 200,000 users [26, 29]. While dating apps, such as Grindr, have allowed SMM to identify romantic and sexual partners, a growing number of research have linked their use to increased body image concerns, unhealthy eating habits, and UWCBs among its users [18, 23, 25]. Many have also attributed dating apps as avenues for body shaming and weight discrimination, prompting certain platforms to ban users who body shame others [30, 31]. Additionally, the prevalence of weight stigma is associated with lowered self-esteem among frequent users of dating apps and may have encouraged SMM to accept a lean and muscular physique as the ideal body type [18, 25]. Thus, SMM engage in UWCBs, such as the use of diet pills, self-induced vomiting, use of muscle-building supplements, anabolic steroids, and fasting, to achieve specific body image ideals [25].

Overall, the available scientific literature on dating app use and its relationship with body image and UWCBs is limited. Only a few studies have examined the association between the use of dating apps and UWCBs [18, 23, 25, 32]. Tran et al. [23], in a study involving 1,769 adult men and women, documented an association between dating app use and elevated rates of UWCBs. This study, however, did not specifically explore the relationship in SMM; it also did not explore the role of body image dissatisfaction in this relationship. Thus, future studies exploring the role of dating app use on body image and UWCBs is warranted. Moreover, research assessing the relationship between dating app use and UWCBs among SMM populations is also sparse. Given the gap in the scientific literature, the present study aims to assess the association between dating app use and UWCBs among adult bisexual and gay men in the U.S. Considering the tripartite influence model, which emphasizes the influence of media on body image dissatisfaction, our second aim is to examine whether controlling for body dissatisfaction affects this association.

## Methods

### Participants

The present study utilized existing data from the Men’s Body Project (MBP) based at the University of New Haven in West Haven, Connecticut. MBP is a cross-sectional study that involves an online survey that assessed various health behaviors (i.e., UWCBs, muscle-building supplement use, steroid use, etc.) and outcomes among bisexual and gay men in the U.S. Participants were invited to participate in an anonymous online survey and were recruited via Qualtrics Survey Panels, which pays them based on a number of factors (e.g., survey length, specific panelist profile, etc.) using cash, airline miles, vouchers, and other incentives. Other epidemiologic surveys have used similar Qualtrics panels [33]. Additional information regarding response screening is provided as supplementary material. Eligible survey participants were those who were: (1) 18–50 years of age, (2) identified as cis-gender gay and bisexual men, (3) English speakers, and (4) residing in the U.S. All participants provided informed consent before participating in the study. The Institutional Review Board of the University of New Haven approved this study (#2022-015).

### Variables measured

**Demographic variables.** The participants provided information on their age (in years), ethnicity, race, sexual orientation, work status, and relationship status.

**Dating app use.** Participants responded to a single item assessing the use of dating apps: *Have you ever used a dating app (example: Tinder, Grindr, Scruff)?* Answer choices included “Yes” or “No.”

**Body image dissatisfaction.** Fifteen items from the Male Body Attitudes Scales (MBAS) were utilized to assess body image dissatisfaction among participants [34]. MBAS is an instrument used to determine body image dissatisfaction among men as it assesses the components - body fat, body height, and muscularity - needed to measure body image dissatisfaction in males [34]. Example items from MBAS include: *I feel embarrassed about my muscularity, I think I have too much fat on my body, I feel ashamed of my height.* A full list of all 15 MBAS items is provided as supplementary material. Response options were based on a six-point scale, (1) Never, (2) Rarely, (3) Sometimes, (4) Often, (5) Usually, (6) Always. Higher scores denote greater body image dissatisfaction [34].

**Unhealthy weight control behaviors and muscle enhancing behaviors.** Participants reported their frequency in engaging in four UWCBs, including fasting for weight control, vomiting for weight control, laxative use, and diet pills use. We also asked other behaviors aimed at changing body shape or size, including use of muscle-building supplements, use of protein powders, and use of anabolic steroids (collectively referred to as muscle enhancing behaviors). Items assessing engagement in UWCBs were adapted from existing questionnaires, including those used as part of the Growing Up Today Study and Youth Risk Behavior Surveillance System [35, 36]. Specifically, our survey asked how frequently, in the past year, participants used diet pills, engaged in fasting (not eating for at least one day), and vomited. Response options include: “Never,” “Less than monthly,” “1–3 times a month,” “Once per week,” “More than once/week.” Participants were also asked about their use of laxatives, anabolic steroids, protein powders, muscle building supplements (creatine, amino acids, DHEA, hydroxyl methyl-butyrate [HMB], or growth hormone), to which they responded using the following response options “Never,” “Less than monthly,” “1–3 times a month,” “Once per week,” “2–6 times per week,” Daily.” All UWCBs, protein powder use, anabolic steroid use, and muscle building supplement use, were split into binary categories for the analysis (“Never vs. all else).

### Analysis

Descriptive statistics, including means, standard deviations, and frequencies, were calculated for demographic variables and total body image dissatisfaction scores. We also compared the distribution of these variables between dating app users and non-users. Chi-square tests and student’s t-test were performed for categorical variables and continuous variables, respectively. These tests were run to evaluate the differences in sociodemographic variables and the UWCBs between users and non-users of dating apps. The association between dating app use and each of the UWCBs, protein powder use, anabolic steroid use, and muscle building supplement use, was examined by performing a series of multivariable logistic regression models. All models controlled for demographic variables included in Table 1. We report odds ratios (OR) and their 95% confidence intervals for logistic regression models with and without the total body image dissatisfaction score separately, referred to as the fully-adjusted and partially-adjusted models, respectively. The tripartite influence model posits that body image dissatisfaction proceeds the onset of eating disorder-related symptoms, including UWCBs [37]. Statistical significance for all analyses was set at p values < 0.05.

**Table 1:** Sociodemographic Characteristics of Dating App Users and Non-Users (N = 549)

Characteristic	App Users (n = 407)	Non-users (n = 142)	Total (N = 549)	p-value
**Age (years), n (%)**				0.169
18 − 15	72 (17.7)	35 (24.6)	107 (19.5)	
25–34	110 (27)	32 (22.5)	142 (25.9)	
35–50	225 (55.3)	75 (52.8)	300 (54.6)	
**Ethnicity, n (%)**				0.776
Hispanic	78 (19.2)	25 (17.6)	103 (18.8)	
Non-Hispanic	329 (80.8)	117 (82.4)	446 (81.2)	
**Race, n (%)**				0.1225
White, Non-Hispanic	301 (74)	91 (64.1)	392 (71.4)	
Black	49 (12)	27 (19)	76 (13.8)	
Asian/Pacific Islander	31 (7.6)	13 (9.2)	44 (8)	
American Indian/Other	26 (6.4)	11 (7.7)	37 (6.7)	
**Sexual Orientation, n (%)**				< 0.001*
Gay	231 (56.8)	55 (38.7)	286 (52.1)	
Bisexual	176 (43.2)	87 (61.3)	263 (47.9)	
**Relationship Status, n (%)**				0.933
Single/Dating	229 (56.3)	78 (54.9)	307 (55.9)	
Living with a partner	57 (14)	23 (16.2)	80 (14.6)	
Married/engaged	102 (25.1)	35 (24.6)	137 (25)	
Divorced/Widowed/Separated/Other	19 (4.7)	6 (4.2)	25 (4.6)	
**Employment Status, n (%)**				0.028*
Full-time	270 (66.3)	77 (54.2)	347 (63.2)	
Part-time	35 (8.6)	14 (9.9)	49 (8.9)	
Student	35 (8.6)	16 (11.3)	51 (9.3)	
Unemployed	56 (13.8)	24 (16.9)	80 (14.6)	
Other	11 (2.7)	11 (7.7)	22 (4)	
**Body Image Dissatisfaction Score**	44.4 (20.2)	37.4 (17.8)	42.6 (19.8)	< 0.001*

*Note.* * Indicates a p-value < 0.05. Chi-Square (χ2) tests and student’s t-test were performed for categorical variables and continuous variables, respectively to examine differences between dating app users and non-users

## Results

### Sociodemographic characteristics

The demographic characteristics of the study sample are described in Table 1. Overall, 549 men participated in the study and were included in these analyses. Nearly 55% (n = 300) were between ages 35–50 years. White participants also represented the majority of the sample (n = 392, 71.4%), followed by Black (n = 76, 13.8%), Asian/Pacific Islander (n = 44, 8%), and American Indian/Other (n = 37, 6.7%). Additionally, slightly more gay men than bisexual men completed the survey (n = 286, 52.1%; n = 263, 47.9%, respectively). The majority of the participants also identified as previous or current users of dating apps (n = 407, 74.1%). Participants that use dating apps were mostly gay men (n = 231, 56.8%) that are dating (n = 229, 56.3%). More than 66% of dating app users were also employed full-time (n = 270). Additionally, dating app users had significantly higher body image dissatisfaction scores than non-users (p < 0.001).

### Prevalence of UWCBs and related behaviors

Table 2 shows the prevalence of UWCBs, protein powder use, anabolic steroid use, and muscle-building supplement use among dating app users versus non-users in the sample. The prevalence of the seven behaviors ranged from 18.8% (n = 103) to 41% (n = 225), with fasting (41%) and protein powder use (39%) among the highest. Results from chi-square tests suggest a significant difference in the prevalence of laxative use, muscle-building supplements, and protein powders between dating app users and non-users; dating app users demonstrated higher prevalence (25.1% vs. 14.1%; 32.2 vs. 17.6%; 42.8% vs. 28.2%) for laxatives, muscle-building supplements, protein powders usage, respectively.

**Table 2:** Unhealthy Weight Control Behaviors and Muscle-Enhancing Behaviors among Dating App Users and Non-Users (N = 549)

UWCB	App users (n = 407)	Non-users (n = 142)	Total (N = 549)	p-value
Fasting, n (%)	170 (41.8)	55 (38.7)	225 (41)	0.237
Vomiting, n (%)	90 (22.1)	25 (17.6)	115 (20.9)	0.103
Laxative use, n (%)	102 (25.1)	20 (14.1)	122 (22.2)	0.037*
Diet pill use, n (%)	105 (25.8)	21 (14.8)	126 (23)	0.082
Use of Muscle-building supplements^a^, n (%)	131 (32.2)	25 (17.6)	156 (28.4)	0.001*
Protein powder use, n (%)	174 (42.8)	40 (28.2)	214 (39)	< 0.001*
Anabolic steroid use, n (%)	82 (20.1)	21 (14.8)	103 (18.8)	0.431

*Note.* * Indicates a p-value < 0.05. Chi-square (χ2) tests and student’s t-test were performed for categorical variables and continuous variables, respectively, to examine differences between dating app users and non-users. ^a^ Includes products such as creatine, amino acids, DHEA, hydroxyl methylbutyrate [HMB], or growth hormone

### Relationship between UWCBs and dating apps use among sexual minority men

Results of our partially- and fully-adjusted logistic regression models are described in Table 3. In our partially-adjusted analyses, dating app users demonstrated significantly higher odds of laxative use (OR = 2.32, 95% CI = 1.319–4.246), diet pill use (OR = 2.21, 95% CI = 1.271–4.004), use of muscle-building supplements (OR = 2.55, 95% CI = 1.527–4.408), and use of protein powders (OR = 2.13, 95% CI = 1.350–3.401) compared to non-users. This association between dating app use and use of laxatives (OR = 2.09, 95% CI = 1.179–3.846), diet pills (OR = 2.00, 95% CI = 1.138–3.633), muscle-building supplements (OR = 2.52, 95% CI = 1.499–4.380), and protein powder (OR = 2.09, 95% CI = 1.321–3.360) remained significant in our fully-adjusted model, which additionally adjusted for participants’ body image dissatisfaction scores.

**Table 3:** Odds of UWCBs and Muscle Enhancing Behaviors in Dating App Users vs. Non-Users

OR (95% CI)		
Behaviors	Partially adjusted^b^	Fully adjusted^c^
Fasting	1.19 (0.781, 1.826)	1.07 (0.699, 1.661)
Vomiting	1.37 (0.792, 2.433)	1.25 (0.718, 2.242)
Laxative use	2.32 (1.319, 4.246)**	2.09 (1.179, 3.846)*
Diet pill use	2.21 (1.271, 4.004)**	2.00 (1.138, 3.633)*
Muscle-building Supplements^a^	2.55 (1.527, 4.408)***	2.52 (1.499, 4.380)***
Protein powder use	2.13 (1.350, 3.401)**	2.09 (1.321, 3.360)**
Anabolic steroid use	1.54 (0.861, 2.833)	1.44 (0.799, 2.671)

*Note.* ***<0.001 **<0.01 *<0.05. OR = Odds Ratios, CI = Confidence Interval

^a^ Includes products such as creatine, amino acids, DHEA, hydroxyl methylbutyrate [HMB], or growth hormone. ^b^Partially-adjusted for demographic characteristics. ^c^Additionally-adjusted for body image dissatisfaction score

## Discussion

Our study’s findings add to the limited scientific literature exploring dating app use among SMM in the U.S. We specifically assessed dating app use and its association with various UWCBs and muscle enhancing behaviors. Considering the tripartite influence model, we originally hypothesized the presence of an association between dating app use and engagement in UWCBs and muscle enhancing behaviors. Our results suggest that dating app use was associated with significantly elevated odds of diet pill use, protein powders, laxatives, and muscle building supplements. Thus, our hypothesis was supported as the prevalence of all the UWCBs were greater among dating app users relative to non-dating app users. However, only four of seven (use of laxatives, diet pills, muscle-building supplements, and protein powders) were significantly associated with dating app use.

The use of dating apps to meet romantic and sexual partners has risen significantly over the years; so has its association with increased body image dissatisfaction, a possible precursor of UWCBs, muscle enhancing behaviors, and the development of eating disorders [18, 25, 31]. Even after accounting for body image dissatisfaction in our models, our results demonstrated significant associations between dating app use and four weight and body shape control behaviors: use of diet pills, protein powders, muscle-building supplements, and laxatives.

Overall, our findings corroborated those of previous studies, but also different in some aspects. In a sample of 1,769 adult men and women, Tran et al. [23] found a significant relationship across six UWCBs examined, including fasting (not eating for at least a day), self-induced vomiting, using laxatives, using diet pills without a doctor’s advice, using anabolic steroids, and using muscle-building supplements (e.g., creatine, amino acids, DHEA, hydroxyl methyl-butyrate [HMB], or growth hormone). Specifically, their study demonstrated dating app users had significantly elevated odds of UWCBs compared to non-users [23]. Our study, on the other hand, documented significant associations between dating app use and four of the seven UWCBs and muscle enhancing behaviors assessed (e.g., laxative use, diet pill use, muscle building supplements, and protein powder use). It should be noted that Tran and colleagues (2019) did not explore protein powder use in their analyses.

Although we did not find significant evidence of a relationship for all seven UWCBs, particularly fasting, vomiting, and anabolic steroid use, the non-significant results from this study do not rule out the possibility of a relationship. Tran et al. [23] focused on adult men and women and did not account for sexual orientation identity, while the present study’s sample was solely comprised of SMM. Thus, there may be underlying cultural differences in weight control and muscle enhancing behaviors between SMM and non-SMM populations that may warrant additional exploration. For instance, studies suggest the desire for muscularity and leanness may differ across sexual orientation identity, such that sexual minority men often strive for more “toned” muscles than their straight male counterparts [38]. It is possible the SMM in our study preferred to engage in some UWCBs and muscle-enhancing behaviors as a result (i.e., avoided the use of anabolic steroids). Furthermore, studies have also documented the role of gender norms influencing weight control behaviors among young men and women [39].

Our findings suggest that SMM that use dating apps may prefer certain UWCBs and muscle enhancing behaviors over others. For instance, dating app users demonstrated elevated use of laxatives, diet pills, protein powders, and muscle-building supplements compared to non-users. Some of these behaviors (e.g., misuse of laxatives) are considered health hazards and are not recommended strategies for weight control [40]. While additional qualitative studies are needed to explore SMM’s attitudes, perceptions, and beliefs around these behaviors, studies have attributed pressure from the media as a driving factor motivating SMM to engage in UWCBs and muscle enhancing behaviors [41]. Online dating apps have many similarities to traditional media in the sense that they also present and promote muscular and lean body types as the socially acceptable physique; many also allow their users to digitally alter their images using key features, such as photo filters and enhancers. The SMM app Grindr was found to encourage appearance comparison and promote weight stigma among its users, which affected users’ body image [42]. Additionally, many, if not most, of these online dating platforms are appearance centered. Thus, they may encourage SMM to compare themselves to others, including those with mesomorphic body types and internalize the body image ideals they see [42]. As a result, SMM may engage in UWCBs and related health risk behaviors with hopes they will achieve the muscular and lean body shape and size they desire.

### Limitations and future directions

While our study yielded innovative results, there are limitations that must be considered. As a cross-sectional study, we cannot establish causal relationships between dating apps and the various UWCBs and muscle enhancing behaviors in this study. For example, it is possible our study participants could have been engaging in UWCBs and muscle enhancing behaviors before the use dating apps. Prior studies suggest UWCBs are quite prevalent among gay and bisexual men of various age groups [14, 43]. Likewise, there might be other extenuating factors that could contribute UWCBs in SMM than dating app use. Our study builds upon previous literature assessing the relationship between dating apps and UWCBs by adjusting for body image dissatisfaction. However, we did not account for participants’ engagement in viewing pornography and other forms of media (e.g., social media, television, etc.). We were also limited to a binary (yes/no) dating app use item, thus, did not account for the frequency of usage in our analyses. A literature review by Parker and Harriger [12] found use of pornography, low self-esteem, high BMI, increased peer pressure to be thin, and internalization of the thin ideal in the gay community to be contributing factors as well. Furthermore, we did not assess participants’ self-esteem, level of internalized homophobia or biphobia, and other psychosocial factors. Thus, future studies should consider the roles of factors that were unmeasured in the dataset analyzed. Additionally, previous studies found bisexual men to be more dissatisfied with their bodies and hence, more likely to engage in UWCBs [18]. We acknowledge that we did not draw comparisons between sexual orientation identity in our sample, especially differences and/or similarities between SMM and their straight male counterparts. In a study involving male participants (SMM and straight participants) from Singapore and Sweden, researchers suggest that sexual orientation identity may moderate the possible relationship between dating app use and body image dissatisfaction [44].

Thus, additional studies assessing dating app use and UWCBs and muscle enhancing behaviors including heterosexual/straight men and SMM are warranted. Furthermore, we mostly focused on the media aspect of the tripartite influence model in this study due to our emphasis on dating app use, which we consider a form of social media. Future studies should also focus on the relationship between media (i.e., dating apps) and the other two components of the model (parents and peers) on SMM’s body image and engagement in UWCBs and muscle enhancing behaviors. Another notable limitation in our study is its generalizability. We are unable to generalize our findings to all populations as we used convenience sampling of SMM through Qualtrics Survey Panels.

Nonetheless, our study adds to the gravely limited literature on the association between dating apps and UWCBs and muscle enhancing behaviors. Our findings not only documented elevated odds of various UWCBs and muscle enhancing behaviors among SMM, but additionally found significantly higher body image dissatisfaction scores among those using dating apps. While we cannot be certain due to the design of our study, we question whether the elevated use of diet pills, protein powders, muscle-building supplements, and laxatives among dating app users is also due to the ease of access, and acceptability and perception of safety of the products associated with these behaviors in SMM communities.

### Implications for Research and Practice

The use of dating apps has increased immensely over the past couple of years in the United States, and so has their link to body image dissatisfaction and UWCBs [18, 23, 45]. UWCBs are considered precursors of the development of eating disorders, such as anorexia nervosa [8, 12, 17, 23]. Additionally, the use of diet pills and laxatives were found to significantly increase the likelihood of people engaging in behaviors such as unprotected sexual activity, substance abuse, and reporting suicidal thoughts [46]. With dating app users in the present study significantly engaging in the use of diet pills and laxatives than non-dating app users, there is a necessity to understand the influence of dating apps on these health risk behaviors. Moreover, our study calls for more research and surveillance of various UWCBs among SMM. Given they are viewed as precursors to the development of eating disorders, it is important that more surveillance is done to track trends in engagement in these behaviors over time.

Furthermore, in response to previous calls, additional research is needed to test the tripartite influence model on diverse populations, as it was originally developed and tested among primarily White samples. Given that our cross-sectional study documented significant associations between dating app use and various UWCBs and muscle enhancing behaviors, even in the absence of body image dissatisfaction in our models, we believe future studies assessing body image disturbance as well as additional constructs of the tripartite influence model (e.g., appearance comparison, media internalization) is warranted.

While it was not a key focus on this study, future research may consider applying the uses and gratifications theory in regard to dating app use. Uses and gratification theory focuses on the premise that individuals seek out media for specific, often personal needs to ultimately achieve gratification [47]. The theory originated from the communications literature and many studies have applied its framework to understand individuals’ motivations for using social media [47, 48]. There is an opportunity to also apply its framework to better understand individuals’ motivations to using dating apps. Prior studies, for example, have assessed the relationship between dating app use and various health behaviors (e.g., smoking), and their findings suggest that such associations may be moderated by individuals’ motivations for using dating apps [49].

Finally, another key implication of our study is the dire need for enhanced screening and detection of UWCBs and eating disorders risk factors in SMM. Such detection is needed at an earlier stage as these behaviors may exacerbate and manifest into eating disorders. Furthermore, studies have documented body image dissatisfaction as a key culprit and motivator for engaging in UWCBs [41]. Thus, interventions must consider addressing concerns around one’s body image dissatisfaction and potential risks of engaging in UWCBs and muscle enhancing behaviors. With our findings suggesting the influence of media on engaging in UWCBs, particularly dating app use, public health and other health officials need to understand the underlying knowledge, beliefs, and perceptions of SMM engaging in UWCBs and muscle enhancing behaviors. Such knowledge may assist in developing tailored approaches to optimizing the health of SMM, including efforts to prevent the development of eating disorders in this historically marginalized population.

## Electronic supplementary material

Below is the link to the electronic supplementary material.

Supplementary Material 1

Supplementary Material 2

## Data Availability

The data generated and analysed during the current study are available from the corresponding author on reasonable request.
