# Supplementary material for: Association between dating app use and unhealthy weight control behaviors and muscle enhancing behaviors in sexual minority men: a cross-sectional study
Source: BMC Public Health. 2023 May 9;23:838. doi: 10.1186/s12889-023-15715-7 (PMC10170774; doi:10.1186/s12889-023-15715-7)
Supplement: Supplementary file 1 — Supplementary Material 1 [file 12889_2023_15715_MOESM1_ESM.docx]

**Supplementary: Brief description of screening protocol for Qualtrics Survey Panels for the Men’s Body Project**

The researchers in this study collaborated with Qualtrics to recruit the participants using its survey panels. According to Qualtrics, participants are recruited from various sources, including website intercept recruitment, member referrals, targeted email lists, gaming sites, customer loyal web portals, permission-based networks, social media, etc. Furthermore, Qualtrics claims to check every IP address, implement digital fingerprinting technology, among other means to exclude duplication and ensure validity. Specifically, for the Men’s Body Project, the data collected and analysed in this study, participants were recruited using enforced screening criteria that reflected the eligibility criteria listed in the methods section. Additionally, completion times of the survey was assessed by the Qualtrics team to automatically exclude respondents who failed a speeding check, measured as one-third the median time to completion of the survey (seven minutes). Lastly, participants who self-reported potentially nonviable or ambiguous weight and height values (e.g., 0 lbs, letters, etc.) were excluded from the final sample.
