# Supplementary material for: Association between dating app use and unhealthy weight control behaviors and muscle enhancing behaviors in sexual minority men: a cross-sectional study
Source: BMC Public Health. 2023 May 9;23:838. doi: 10.1186/s12889-023-15715-7 (PMC10170774; doi:10.1186/s12889-023-15715-7)
Supplement: Supplementary file 2 — Supplementary Material 2 [file 12889_2023_15715_MOESM2_ESM.docx]

**Supplementary: 15 Item Male Body Attitudes Scale (MBAS) items**

1. I think I have too little muscle on my body
   1. Never
   2. Rarely
   3. Sometimes
   4. Often
   5. Usually
   6. Always
2. I think my legs are not muscular enough
   1. Never
   2. Rarely
   3. Sometimes
   4. Often
   5. Usually
   6. Always
3. I think my arms should be more muscular
4. Never
5. Rarely
6. Sometimes
7. Often
8. Usually
9. Always
10. I feel embarrassed about my muscularity
    1. Usually
    2. Always
    3. Never
    4. Rarely
    5. Sometimes
    6. Often
11. I think my back should be more muscular
    1. Usually
    2. Always
    3. Never
    4. Rarely
    5. Sometimes
    6. Often
12. I think my chest should be more muscular
13. Usually
14. Always
15. Never
16. Rarely
17. Sometimes
18. Often
19. I feel satisfied with my muscularity
    1. Always
    2. Usually
    3. Often
    4. Sometimes
    5. Rarely
    6. Never
20. I think my body should be leaner
    1. Always
    2. Usually
    3. Often
    4. Sometimes
    5. Rarely
    6. Never
21. I think I have too much fat on my body
    1. Rarely
    2. Never
    3. Always
    4. Usually
    5. Often
    6. Sometimes
22. Eating sweets, cakes, or other high calorie food makes me feel fat
    1. Rarely
    2. Never
    3. Always
    4. Usually
    5. Often
    6. Sometimes
23. I feel excessively fat
    1. Rarely
    2. Never
    3. Always
    4. Usually
    5. Often
    6. Sometimes
24. Seeing my reflection (e.g., in a mirror or window) makes me feel badly about my body fat
    1. Never
    2. Rarely
    3. Sometimes
    4. Often
    5. Usually
    6. Always
25. I wish I were taller
    1. Never
    2. Rarely
    3. Sometimes
    4. Often
    5. Usually
    6. Always
26. I am satisfied with my height
    1. Sometimes
    2. Rarely
    3. Never
    4. Always
    5. Usually
    6. Often
27. I feel ashamed of my height
    1. Sometimes
    2. Rarely
    3. Never
    4. Always
    5. Usually
    6. Often
